# Supplementary material for: TransMarker: Unveiling dynamic network biomarkers in cancer progression through cross-state graph alignment and optimal transport
Source: PLoS Comput Biol. 2025 Nov 24;21(11):e1013743. doi: 10.1371/journal.pcbi.1013743 (PMC12668635; doi:10.1371/journal.pcbi.1013743)
Supplement: S2 Table — Performance outcomes for all metrics across the complete range of α values utilized in the Topological Feature Integration step. (PDF) [file pcbi.1013743.s007.pdf]

**Table S2. Detailed evaluation metrics for various $\alpha$ values.** Performance outcomes for all metrics across the complete range of $\alpha$ values utilized in the Topological Feature Integration step.

| $\alpha$ | Accuracy | AUROC | AUPRC | F1 Score | Precision | Recall | Specificity |
|---|---|---|---|---|---|---|---|
| 0.1 | 0.8156 ± 0.0489 | 0.8896 ± 0.0477 | 0.8028 ± 0.0375 | 0.7423 ± 0.0415 | 0.7477 ± 0.0411 | 0.7472 ± 0.0420 | 0.8698 ± 0.0715 |
| 0.2 | 0.8478 ± 0.0722 | 0.9205 ± 0.0167 | 0.8015 ± 0.0355 | 0.7363 ± 0.0433 | 0.7425 ± 0.0421 | 0.7413 ± 0.0446 | 0.8772 ± 0.0547 |
| 0.3 | **0.8755 ± 0.0341** | **0.9230 ± 0.0385** | **0.8871 ± 0.0413** | **0.8607 ± 0.0357** | **0.8688 ± 0.0556** | **0.8686 ± 0.0286** | 0.8808 ± 0.0531 |
| 0.4 | 0.8240 ± 0.0691 | 0.9214 ± 0.0158 | 0.8066 ± 0.0364 | 0.7927 ± 0.0530 | 0.7865 ± 0.0559 | 0.7931 ± 0.0610 | **0.8944 ± 0.0380** |
| 0.5 | 0.7924 ± 0.0749 | 0.8948 ± 0.0434 | 0.7753 ± 0.0675 | 0.7158 ± 0.0486 | 0.7113 ± 0.0561 | 0.7284 ± 0.0547 | 0.8794 ± 0.0555 |
| 0.6 | 0.7281 ± 0.0780 | 0.8943 ± 0.0363 | 0.7909 ± 0.0655 | 0.7111 ± 0.0830 | 0.7303 ± 0.0828 | 0.7230 ± 0.0834 | 0.8740 ± 0.0403 |
| 0.7 | 0.7018 ± 0.0807 | 0.7746 ± 0.0871 | 0.7398 ± 0.0965 | 0.6864 ± 0.0832 | 0.7230 ± 0.0753 | 0.7249 ± 0.0613 | 0.7780 ± 0.1297 |
| 0.8 | 0.7148 ± 0.0548 | 0.7332 ± 0.0745 | 0.7553 ± 0.0631 | 0.7079 ± 0.0377 | 0.7291 ± 0.0409 | 0.7381 ± 0.0400 | 0.8007 ± 0.0659 |
| 0.9 | 0.6799 ± 0.0696 | 0.7256 ± 0.0644 | 0.7267 ± 0.0552 | 0.7128 ± 0.0512 | 0.7192 ± 0.0520 | 0.7141 ± 0.0483 | 0.7987 ± 0.0928 |
