# Supplementary material for: TransMarker: Unveiling dynamic network biomarkers in cancer progression through cross-state graph alignment and optimal transport
Source: PLoS Comput Biol. 2025 Nov 24;21(11):e1013743. doi: 10.1371/journal.pcbi.1013743 (PMC12668635; doi:10.1371/journal.pcbi.1013743)
Supplement: S3 Table — It enumerates average performance scores for each metric across varying training set sizes (10%–50%). (PDF) [file pcbi.1013743.s008.pdf]

S3 Table. Summary of evaluation metrics across different training data proportions. It enumerates average performance scores for each metric across varying training set sizes (10%–50%).

**Test**	**(10%)**	**(20%)**	**(30%)**	**(40%)**	**(50%)**
Accuracy	0.8750 ± 0.0886	**0.8755 ± 0.0341**	0.8134 ± 0.0669	0.7610 ± 0.0642	0.7365 ± 0.0430
AUROC	0.9143 ± 0.0206	**0.9230 ± 0.0385**	0.9059 ± 0.0112	0.8828 ± 0.0741	0.8649 ± 0.1116
AUPRC	0.8034 ± 0.0374	**0.8871 ± 0.0413**	0.8021 ± 0.0381	0.8023 ± 0.0361	0.8317 ± 0.0583
F1 Score	0.7308 ± 0.0426	**0.8607 ± 0.0357**	0.7339 ± 0.0439	0.7258 ± 0.0416	0.7403 ± 0.0361
Precision	0.7372 ± 0.0414	**0.8688 ± 0.0556**	0.7385 ± 0.0430	0.7184 ± 0.0476	0.7457 ± 0.0354
Recall	0.7365 ± 0.0417	**0.8686 ± 0.0286**	0.7391 ± 0.0444	0.7141 ± 0.0544	0.7456 ± 0.0363
Specificity	0.8492 ± 0.0720	**0.8808 ± 0.0531**	0.8395 ± 0.0562	0.8196 ± 0.0996	0.8073 ± 0.0869
