# Supplementary material for: TransMarker: Unveiling dynamic network biomarkers in cancer progression through cross-state graph alignment and optimal transport
Source: PLoS Comput Biol. 2025 Nov 24;21(11):e1013743. doi: 10.1371/journal.pcbi.1013743 (PMC12668635; doi:10.1371/journal.pcbi.1013743)
Supplement: S1 Text — (PDF) [file pcbi.1013743.s009.pdf]

S1. Details of Highly Related Methods and Evaluation Metrics

To benchmark the effectiveness of our proposed framework in identifying DNBs across disease progression states, we compared it with five state-of-the-art multilayer network ranking methods. These methods capture diverse aspects of node importance across multilayer topologies and serve as robust baselines for multilayer network analysis.

Multilayer entropy [8]: The method, which introduces a new centrality ranking approach for multilayer networks, addresses the limitations of single-layer centrality measures by incorporating both within-layer and between-layer structural information. It builds upon traditional centrality metrics, such as betweenness centrality, by considering a node's influence across layers. In particular, it assigns higher scores to nodes that connect multiple layers or act as bridges between them. Using these adjusted scores along with node degrees, the method computes a weighted local structure entropy to quantify each node's informational contribution. By aggregating entropy values across all layers, the approach yields a comprehensive ranking that captures both local and global importance, enhancing accuracy and reducing information loss—especially in large or complex multilayer networks.

On the GAC dataset, multilayer-entropy achieved an AUROC of 0.8319, but yielded low classification performance with an accuracy of 0.5653, AUPRC of 0.5121, and F1 score of 0.4983. On the ESCC dataset, performance declined further: Accuracy of 0.4904, AUROC of 0.7911, and AUPRC of 0.5145, indicating its inability to prioritize disease-relevant genes reliably in these settings.

ElementRank [9]: This method represents a multilayer ranking approach that extends PageRank to complex network structures. In its framework, a weighted PageRank score is firstly computed independently for each layer of the network. To account for the varying significance of different layers, each layer's PageRank results are then scaled by a weight reflecting its relative importance—these weights can be derived from domain expertise or techniques such as the Analytic Hierarchy Process (AHP). The final global score for each node is obtained by aggregating its layer-specific scores through a weighted average. Additionally, a virtual "ground node" is introduced within each layer to ensure strong connectivity, which enhances the stability and convergence of the PageRank algorithm across all layers. This approach allows for a balanced and layer-aware assessment of node importance in multilayer networks.

For GAC, ElementRank slightly outperformed multilayer-entropy in AUROC (0.8519) and AUPRC (0.5415), but still showed low accuracy (0.5626) and F1 score (0.5024). In ESCC, it achieved an accuracy of 0.5252, AUROC of 0.8001, and AUPRC of 0.5567, highlighting marginal improvement over multilayer entropy but still lagging behind in overall effectiveness.

Versatility Centrality [10]: Traditional centrality measures like eigenvector centrality and PageRank are limited in their ability to reflect a node's influence in interconnected multilayer networks. To address this, the versatility centrality method introduces a tensor-based approach that evaluates a node's overall centrality across all layers. The process involves computing eigenvector centrality for each node within each individual layer, forming a centrality vector that spans layers. The final versatility score is calculated as the L2 norm of this vector, optionally normalized by the number of layers in which the node is active. This formulation captures both the magnitude and consistency of a node's central role throughout the multilayer structure.

In GAC, versatility centrality performed poorly with an accuracy of 0.4587, AUROC of 0.7611, and AUPRC of 0.4028, making it the weakest performer among all methods. This trend persisted in ESCC, where it achieved accuracy of 0.4012, AUROC of 0.6943, and AUPRC of 0.4014, indicating limited utility in disease gene prioritization tasks.

Versatility Degree Centrality [11]: This method prioritizes nodes in multilayer biological networks, such as those constructed from multi-omics data like gene expression and methylation. It assesses each node's connectivity within individual layers and then aggregates this information to determine its overall importance across the network. By considering both the total number of connections a node has and the number of layers in which it is active, the method highlights nodes that are consistently influential, especially those that show strong connections in fewer layers—indicating specialized or cross-layer relevance. An optional filtering step based on statistical thresholds can be applied to identify the most prominent nodes.

For GAC, versatility degree yielded improved performance: Accuracy of 0.6453, AUROC of 0.8693, and AUPRC of 0.6069. On ESCC, it also performed better than most baselines, with Accuracy of 0.5915, AUROC of 0.8375, and AUPRC of 0.6099, ranking second among all comparison methods.

Eigenvector Multicentrality [12]: This approach extends eigenvector centrality to the context of multilayer networks using a tensor-based model. It represents the network structure with a high-order adjacency tensor and integrates interlayer influence through an additional tensor. The resulting

multicentrality score for each node reflects its importance within layers as well as across layers, weighted by the significance of each layer. The scores are computed by applying a power iteration algorithm to derive the dominant eigenvector of the adjusted interaction tensor.

For GAC, it achieved Accuracy of 0.4338, AUROC of 0.8019, and AUPRC of 0.5035. On ESCC, results were similar: Accuracy of 0.4432, AUROC of 0.8256, and AUPRC of 0.4967, showing that while the theoretical model captures multilayer structure, its practical prioritization performance is limited.

We evaluated the performance of our proposed method and all baseline models using the following standard classification metrics, which provide insight into various aspects of predictive accuracy and reliability in the context of disease state classification.

- **ACC:** Measures the overall correctness of the classifier by computing the proportion of true predictions (both positive and negative) among all predictions. In our study, a high accuracy indicates that the method effectively distinguishes state-specific biomarkers from non-relevant genes.
- **AUROC:** Captures the model's ability to distinguish between classes across all thresholds. A higher AUROC score indicates that the method can robustly separate disease-associated genes across multiple states with high confidence.
- **AUPRC:** Especially useful in imbalanced datasets, AUPRC quantifies how many of the predicted positive associations (e.g., state-specific genes) are truly relevant. A high AUPRC value reflects the model's strength in retrieving high-confidence state biomarkers.
- **F1 Score:** The harmonic mean of precision and recall. It evaluates the model's balance between false positives and false negatives. A higher F1 score in our study confirms that the predicted gene sets are not only accurate but also consistent across states.
- **Precision:** The proportion of true positives among all predicted positives. In this context, precision reflects how reliably our method identifies disease-relevant genes without including irrelevant ones.
- **Recall (Sensitivity):** Measures the proportion of true state-specific biomarkers correctly identified by the method. High recall values indicate the model's robustness in capturing known and novel relevant genes.
- **Specificity:** Reflects the method's ability to correctly reject non-disease genes. High specificity is particularly important for reducing false positives in biomarker discovery.

The mathematical formulations of these metrics are provided below:

$$\text{Accuracy (ACC)} = \frac{TP + TN}{TP + TN + FP + FN}$$
$$\text{Precision} = \frac{TP}{TP + FP}$$
$$\text{Recall} = \frac{TP}{TP + FN}$$
$$\text{F1 Score} = 2 \times \frac{\text{Precision} \times \text{Recall}}{\text{Precision} + \text{Recall}}$$
$$\text{Specificity} = \frac{TN}{TN + FP}$$

The ROC curve is generated using:

$$\text{TPR} = \frac{TP}{TP + FN}, \quad \text{FPR} = \frac{FP}{FP + TN}$$

The Precision-Recall (PR) curve is computed based on Precision and Recall, where TP = True Positives, TN = True Negatives, FP = False Positives, and FN = False Negatives.

Across both GAC and ESCC datasets, our method achieved the highest performance in nearly all evaluation metrics compared to the five state-of-the-art multilayer network ranking approaches. This comprehensive advantage demonstrates the effectiveness of our model's integration of dynamic expression signals, topological features, and state-specific rewiring mechanisms, thereby establishing it as a powerful and generalizable approach for multilayer biomarker prioritization.

References

[1] Wang R, Song S, Qin J, et al. Evolution of immune and stromal cell states and ecotypes during gastric adenocarcinoma progression. Cancer Cell. 2023;41(8):1407-1426.e9.

[2] Zhang P, Yang M, Zhang Y, et al. Dissecting the Single-Cell Transcriptome Network Underlying Gastric Premalignant Lesions and Early Gastric Cancer. Cell Rep. 2019;27(6):1934-1947.e5.

[3] Sathe A, Grimes SM, Lau BT, et al. Single-Cell Genomic Characterization Reveals the Cellular Reprogramming of the Gastric Tumor Microenvironment. Clin Cancer Res. 2020;26(11):2640-2653.

[4] Liu T, Zhao X, Lin Y, et al. Computational identification of preneoplastic cells displaying high stemness and risk of cancer progression. *Cancer Res.* 2022;82(14):2520–2537.

[5] Liu ZP, Wu C, Miao H, et al. RegNetwork: an integrated database of transcriptional and post-transcriptional regulatory networks in human and mouse. Database. 2015;2015:bav095.

[6] Dibaeinia P, Sinha S. SERGIO: A Single-Cell Expression Simulator Guided by Gene Regulatory Networks. Cell Syst. 2020;11(3):252-271.e11.

[7] Butler A, Hoffman P, Smibert P, et al. Integrating single-cell transcriptomic data across different conditions, technologies, and species. Nat Biotechnol. 2018;36(5):411-420.

[8] Wang D, Tian F, Wei D. A new centrality ranking method for multilayer networks. Journal of Computational Science. 2023;66:101924.

[9] Pan W, Ming H, Chang CK, et al. ElementRank: Ranking java software classes and packages using a multilayer complex network-based approach. IEEE Transactions on Software Engineering. 2019;47(10):2272–2295.

[10] De Domenico M, Solé-Ribalta A, Omodei E, et al. Ranking in interconnected multilayer networks reveals versatile nodes. Nature Communications. 2015;6(1):6868.

[11] Saha S, Bandyopadhyay S. Versatility-preserving multi-omics data analysis by ranking the nodes in multilayer network. In: 2020 IEEE 5th International Conference on Computing Communication and Automation (ICCCA). 2020;617–622.

[12] Wu M, He S, Zhang Y, et al. A tensor-based framework for studying eigenvector multicentrality in multilayer networks. Proceedings of the National Academy of Sciences. 2019;116(31):15407–15413.

[13] Brandes U. On variants of shortest-path betweenness centrality and their generic computation. Social Networks. 2008;30:136–145.

[14] Faghani MR, Nguyen UT. A study of XSS worm propagation and detection mechanisms in online social networks. IEEE Transactions on Information Forensics and Security. 2013;8:1815–1826.

[15] Freeman LC. Centrality in social networks conceptual clarification. Social Networks. 1978;1:215–239.

[16] Pal SK, Kundu S, Murthy CA. Centrality measures, upper bound, and influence maximization in large scale directed social networks. Fundamenta Informaticae. 2014;130:317–342.

[17] Opsahl T, Agneessens F, Skvoretz J. Node centrality in weighted networks: generalizing degree and shortest paths. Social Networks. 2010;32:245–251.

[18] Boldi P, Vigna S. Axioms for centrality. Internet Mathematics. 2014;10:222–262.

[19] Qi X, Fuller E, Wu Q, et al. Laplacian centrality: a new centrality measure for weighted networks. Information Sciences. 2012;194:240–253.

[20] Chen D, Lü L, Shang MS, et al. Identifying influential nodes in complex networks. Physica A. 2012;391:1777–1787.

[21] Lu L, Zhang YC, Yeung CH, et al. Leaders in social networks, the Delicious case. PLoS One. 2011;6:e21202.

[22] Joyce KE, Laurienti PJ, Burdette JH, et al. A new measure of centrality for brain networks. PLoS One. 2010;5:e12200.

[23] Dangalchev C. Residual closeness in networks. Physica A. 2006;365:556–564.

[24] Valente TW, Foreman RK. Integration and radiality: measuring the extent of an individual's connectedness and reachability in a network. Social Networks. 1998;20:89–105.

[25] Page L, Brin S, Motwani R, et al. The PageRank Citation Ranking: Bringing Order to the Web. Stanford InfoLab. 1999.

[26] Kim CY, Baek S, Cha J, Yang S, Kim E, Marcotte EM, Hart T, Lee I. HumanNet v3: an improved database of human gene networks for disease research. Nucleic Acids Research. 2022;50(D1):D632–D639.

[27] Lage K, Karlberg EO, Størling ZM, Olason PI, Pedersen AG, Rigina O, Hinsby AM, Tümer Z, Pociot F, Tommerup N, et al. A human phenome–interactome network of protein complexes implicated in genetic disorders. Nature Biotechnology. 2007;25(3):309–316.

[28] Szklarczyk D, Gable AL, Lyon D, Junge A, Wyder S, Huerta-Cepas J, Simonovic M, Doncheva NT, Morris JH, Bork P, et al. STRING v11: protein–protein association networks with increased coverage, supporting functional discovery in genome-wide experimental datasets. Nucleic Acids Research. 2019;47(D1):D607–D613.
