# Supplementary material for: TransMarker: Unveiling dynamic network biomarkers in cancer progression through cross-state graph alignment and optimal transport
Source: PLoS Comput Biol. 2025 Nov 24;21(11):e1013743. doi: 10.1371/journal.pcbi.1013743 (PMC12668635; doi:10.1371/journal.pcbi.1013743)
Supplement: S4 Table — Evaluation encompasses accuracy, AUROC, AUPRC, and other standard metrics computed over 50 independent runs. (PDF) [file pcbi.1013743.s010.pdf]

Table S4. Summary of performance metrics for our method and 13 centrality-based node ranking methods on the GAC dataset. Evaluation encompasses accuracy, AUROC, AUPRC, and other standard metrics computed over 50 independent runs.

Method	Accuracy	AUROC	AUPRC	F1 Score	Precision	Recall	Specificity
TransMarker	0.8755 ± 0.0341	0.9230 ± 0.0385	0.8871 ± 0.0413	0.8607 ± 0.0357	0.8688 ± 0.0556	0.8686 ± 0.0286	0.8808 ± 0.0531
Betweenness	0.5858 ± 0.0851	0.7256 ± 0.0457	0.4926 ± 0.0456	0.4900 ± 0.0343	0.4822 ± 0.0212	0.5202 ± 0.0094	0.7919 ± 0.0432
Bottleneck	0.5400 ± 0.0321	0.7220 ± 0.0425	0.4874 ± 0.0422	0.4748 ± 0.0124	0.4841 ± 0.0322	0.5405 ± 0.0213	0.7860 ± 0.0234
Degree	0.6049 ± 0.0633	0.7389 ± 0.0522	0.5322 ± 0.0133	0.5118 ± 0.0213	0.5011 ± 0.0432	0.5461 ± 0.0237	0.7967 ± 0.0144
Diffusion	0.6048 ± 0.0186	0.7607 ± 0.0244	0.5598 ± 0.0322	0.5192 ± 0.0165	0.5183 ± 0.0433	0.5614 ± 0.0255	0.7989 ± 0.0156
Latora	0.3987 ± 0.0347	0.6786 ± 0.0412	0.4264 ± 0.0352	0.3637 ± 0.0321	0.4016 ± 0.0443	0.4689 ± 0.0145	0.7558 ± 0.0093
Lin	0.5707 ± 0.0175	0.7302 ± 0.0096	0.5462 ± 0.0432	0.4836 ± 0.0366	0.4778 ± 0.0098	0.5281 ± 0.0134	0.7847 ± 0.0165
Laplacian	0.5893 ± 0.0214	0.7286 ± 0.0644	0.5101 ± 0.0231	0.4956 ± 0.0538	0.4901 ± 0.0034	0.5298 ± 0.0145	0.7940 ± 0.0154
Local	0.5627 ± 0.0285	0.7241 ± 0.0335	0.5322 ± 0.0331	0.5136 ± 0.0264	0.5080 ± 0.0473	0.5285 ± 0.0134	0.7792 ± 0.0034
Leaderrank	0.5987 ± 0.0186	0.7371 ± 0.0535	0.5243 ± 0.0321	0.5072 ± 0.0218	0.5095 ± 0.0754	0.5564 ± 0.0134	0.7960 ± 0.0323
Leverage	0.5667 ± 0.0013	0.7386 ± 0.0467	0.5342 ± 0.0094	0.4991 ± 0.0432	0.5210 ± 0.0763	0.5638 ± 0.0145	0.7933 ± 0.0345
Residual	0.5707 ± 0.0344	0.7626 ± 0.0105	0.5831 ± 0.0176	0.5394 ± 0.0325	0.5351 ± 0.0343	0.6023 ± 0.0133	0.7899 ± 0.0324
Radiality	0.4218 ± 0.0633	0.6967 ± 0.0246	0.4741 ± 0.0326	0.3890 ± 0.0214	0.4154 ± 0.0365	0.4760 ± 0.0233	0.7589 ± 0.0265
Pagerank	0.6338 ± 0.0415	0.8045 ± 0.0136	0.5486 ± 0.0124	0.4799 ± 0.0157	0.5018 ± 0.0301	0.5732 ± 0.0324	0.8871 ± 0.0910
