# Supplementary material for: TransMarker: Unveiling dynamic network biomarkers in cancer progression through cross-state graph alignment and optimal transport
Source: PLoS Comput Biol. 2025 Nov 24;21(11):e1013743. doi: 10.1371/journal.pcbi.1013743 (PMC12668635; doi:10.1371/journal.pcbi.1013743)
Supplement: S5 Table — (PDF) [file pcbi.1013743.s012.pdf]

Table S5. List of 13 centrality methods utilized for comparative study.

#	Method	Formula	Reference
1	Betweenness Centrality	$BC(v) = \sum_{s,t} \frac{\sigma(s,t\|v)}{\sigma(s,t)}$	[13]
2	Bottleneck Centrality	$BN(v) = \sum_{S\in V} P_s(v)$	[14]
3	Degree Centrality	$d(v) = \frac{k_v}{N-1}$	[15]
4	Diffusion Degree	$C_d(v) = \lambda_v C_D(v) + \sum_{i\in\text{neighbors}} \lambda_i C_D(v_i)$	[16]
5	Latora Closeness Centrality	$C_c^*(v) = \frac{1}{\sum_{i\neq v} d(v,i)}$	[17]
6	Lin Centrality	$C_{\text{Lin}(x)} = \frac{\|\{y\|d(x,y)<\infty\}\|^2}{\sum_{d(x,y)<\infty} d(x,y)}$	[18]
7	Laplacian Centrality	$C_v^L = d_G^2(v) + d_G(v) + 2\sum_{v_i\in N(v)} d_G(v_i)$	[19]
8	Local Centrality	$C_L(v) = \sum_{u\in\tau(v)} Q(u)$	[20]
9	LeaderRank	$S_i = s_i(t_c) + \frac{s_g(t_c)}{N}, \quad s_j(t+1) = \sum_{j=1}^{N+1} \frac{a_{ji}}{k_j^{out}} s_j(t)$	[21]
10	Leverage Centrality	$l_i = \frac{1}{k_i}\sum_{N_i} \frac{k_i - k_j}{k_i + k_j}$	[22]
11	Residual Closeness Centrality	$C_k = \sum_i \sum_{j\neq i} \frac{1}{2^{d_k(i,j)}}$	[23]
12	Radiality Centrality	$C_{\text{rad}}(v) = \frac{\sum_{w\in V}(\Delta G + 1 - \text{dist}(v,w))}{n-1}$	[24]
13	Pagerank	$PR(i) = \frac{1-d}{N} + d\sum_{j\in N_i} \frac{PR(i)}{k_j^{out}}$	[25]

References

[1] Wang R, Song S, Qin J, et al. Evolution of immune and stromal cell states and ecotypes during gastric adenocarcinoma progression. Cancer Cell. 2023;41(8):1407-1426.e9.

[2] Zhang P, Yang M, Zhang Y, et al. Dissecting the Single-Cell Transcriptome Network Underlying Gastric Premalignant Lesions and Early Gastric Cancer. Cell Rep. 2019;27(6):1934-1947.e5.

[3] Sathe A, Grimes SM, Lau BT, et al. Single-Cell Genomic Characterization Reveals the Cellular Reprogramming of the Gastric Tumor Microenvironment. Clin Cancer Res. 2020;26(11):2640-2653.

[4] Liu T, Zhao X, Lin Y, et al. Computational identification of preneoplastic cells displaying high stemness and risk of cancer progression. *Cancer Res.* 2022;82(14):2520–2537.

[5] Liu ZP, Wu C, Miao H, et al. RegNetwork: an integrated database of transcriptional and post-transcriptional regulatory networks in human and mouse. Database. 2015;2015:bav095.

[6] Dibaeinia P, Sinha S. SERGIO: A Single-Cell Expression Simulator Guided by Gene Regulatory Networks. Cell Syst. 2020;11(3):252-271.e11.

[7] Butler A, Hoffman P, Smibert P, et al. Integrating single-cell transcriptomic data across different conditions, technologies, and species. Nat Biotechnol. 2018;36(5):411-420.

[8] Wang D, Tian F, Wei D. A new centrality ranking method for multilayer networks. Journal of Computational Science. 2023;66:101924.

[9] Pan W, Ming H, Chang CK, et al. ElementRank: Ranking java software classes and packages using a multilayer complex network-based approach. IEEE Transactions on Software Engineering. 2019;47(10):2272–2295.

[10] De Domenico M, Solé-Ribalta A, Omodei E, et al. Ranking in interconnected multilayer networks reveals versatile nodes. Nature Communications. 2015;6(1):6868.

[11] Saha S, Bandyopadhyay S. Versatility-preserving multi-omics data analysis by ranking the nodes in multilayer network. In: 2020 IEEE 5th International Conference on Computing Communication and Automation (ICCCA). 2020;617–622.

[12] Wu M, He S, Zhang Y, et al. A tensor-based framework for studying eigenvector multicentrality in multilayer networks. Proceedings of the National Academy of Sciences. 2019;116(31):15407–15413.

[13] Brandes U. On variants of shortest-path betweenness centrality and their generic computation. Social Networks. 2008;30:136–145.

[14] Faghani MR, Nguyen UT. A study of XSS worm propagation and detection mechanisms in online social networks. IEEE Transactions on Information Forensics and Security. 2013;8:1815–1826.

[15] Freeman LC. Centrality in social networks conceptual clarification. Social Networks. 1978;1:215–239.

[16] Pal SK, Kundu S, Murthy CA. Centrality measures, upper bound, and influence maximization in large scale directed social networks. Fundamenta Informaticae. 2014;130:317–342.

[17] Opsahl T, Agneessens F, Skvoretz J. Node centrality in weighted networks: generalizing degree and shortest paths. Social Networks. 2010;32:245–251.

[18] Boldi P, Vigna S. Axioms for centrality. Internet Mathematics. 2014;10:222–262.

[19] Qi X, Fuller E, Wu Q, et al. Laplacian centrality: a new centrality measure for weighted networks. Information Sciences. 2012;194:240–253.

[20] Chen D, Lü L, Shang MS, et al. Identifying influential nodes in complex networks. Physica A. 2012;391:1777–1787.

[21] Lu L, Zhang YC, Yeung CH, et al. Leaders in social networks, the Delicious case. PLoS One. 2011;6:e21202.

[22] Joyce KE, Laurienti PJ, Burdette JH, et al. A new measure of centrality for brain networks. PLoS One. 2010;5:e12200.

[23] Dangalchev C. Residual closeness in networks. Physica A. 2006;365:556–564.

[24] Valente TW, Foreman RK. Integration and radiality: measuring the extent of an individual's connectedness and reachability in a network. Social Networks. 1998;20:89–105.

[25] Page L, Brin S, Motwani R, et al. The PageRank Citation Ranking: Bringing Order to the Web. Stanford InfoLab. 1999.

[26] Kim CY, Baek S, Cha J, Yang S, Kim E, Marcotte EM, Hart T, Lee I. HumanNet v3: an improved database of human gene networks for disease research. Nucleic Acids Research. 2022;50(D1):D632–D639.

[27] Lage K, Karlberg EO, Størling ZM, Olason PI, Pedersen AG, Rigina O, Hinsby AM, Tümer Z, Pociot F, Tommerup N, et al. A human phenome–interactome network of protein complexes implicated in genetic disorders. Nature Biotechnology. 2007;25(3):309–316.

[28] Szklarczyk D, Gable AL, Lyon D, Junge A, Wyder S, Huerta-Cepas J, Simonovic M, Doncheva NT, Morris JH, Bork P, et al. STRING v11: protein–protein association networks with increased coverage, supporting functional discovery in genome-wide experimental datasets. Nucleic Acids Research. 2019;47(D1):D607–D613.
