# Supplementary material for: TransMarker: Unveiling dynamic network biomarkers in cancer progression through cross-state graph alignment and optimal transport
Source: PLoS Comput Biol. 2025 Nov 24;21(11):e1013743. doi: 10.1371/journal.pcbi.1013743 (PMC12668635; doi:10.1371/journal.pcbi.1013743)
Supplement: S6 Table — (PDF) [file pcbi.1013743.s013.pdf]

**S6 Table. The dynamic network biomarkers based on TransMarker, a long with the summaries of their functions.**

| Gene symbol | Gene name | Known functions |
|---|---|---|
| GSPT1 | G1 To S Phase Transition 1 | Involved in regulation of translational termination and translational termination. |
| GAB1 | GRB2 Associated Binding Protein 1 | Plays a central role in cellular growth response, transformation and apoptosis. |
| FZD7 | Frizzled Class Receptor 7 | Receptor for Wnt proteins. |
| TFF3 | Trefoil Factor 3 | Protects the mucosa from insults, stabilizes the mucus layer and affects healing of the epithelium. |
| BCL2 | BCL2 Apoptosis Regulator | Blocks the apoptotic death of some cells such as lymphocytes. |
| FZD5 | Frizzled Class Receptor 5 | Functions in the canonical Wnt/$\beta$-catenin signaling pathway. |
| MAPK3 | Mitogen-Activated Protein Kinase 3 | Regulates cell proliferation, differentiation, and survival. |
| MAPK1 | Mitogen-Activated Protein Kinase 1 | Mediates diverse biological functions such as cell growth, adhesion, survival and differentiation through regulation of transcription, translation, and cytoskeletal rearrangements. |
| LRP6 | LDL Receptor Related Protein 6 | Acts as an essential co-receptor of Wnt/$\beta$-catenin signaling. |
| TCF7 | Transcription Factor 7 | Plays a critical role in natural killer cell and innate lymphoid cell development. |
| KLF4 | KLF Transcription Factor 4 | Regulates the expression of key transcription factors during embryonic development. |
| CDH17 | Cadherin 17 | Is a useful diagnostic marker for adenocarcinomas of the digestive system. |
| ITK | IL2 Inducible T Cell Kinase | Tyrosine kinase essential for regulation of the adaptive immune response. |
| FZD10 | Frizzled Class Receptor 10 | Receptor for Wnt proteins. |
| IL8 | C-X-C Motif Chemokine Ligand 8 | Chemotactic factor that mediates inflammatory response by attracting neutrophils, basophils, and T-cells. |
| ETS2 | ETS Proto-Oncogene 2 | Encodes a transcription factor regulating genes involved in development and apoptosis. |
| CDX2 | Caudal Type Homeobox 2 | Major regulator of intestine-specific genes involved in cell growth and differentiation. |
| CDKN1B | Cyclin Dependent Kinase Inhibitor 1B | Binds to and prevents activation of cyclin E-CDK2 or cyclin D-CDK4 complexes, controlling cell cycle at G1. |
| GNB2L1 | Receptor For Activated C Kinase 1 | Enables cyclin binding activity, enzyme binding activity, and protein domain specific binding activity. |
| STAT1 | Signal Transducer And Activator Of Transcription 1 | Mediates cellular responses to interferons (IFNs), cytokines and other growth factors. |
| CTNNA3 | Catenin Alpha 3 | Plays a role in cell-cell adhesion in muscle cells. |
| PIK3CA | Phosphatidylinositol-4,5-Bisphosphate 3-Kinase Catalytic Subunit Alpha | Oncogene with hotspots at helical and kinase domains. |
| TGFBR2 | Transforming Growth Factor Beta Receptor 2 | Regulates transcription of genes related to proliferation, cell cycle arrest, wound healing, immunosuppression, and tumorigenesis. |

**S6 Table. The dynamic network biomarkers based on TransMarker, a long with the summaries of their functions.**

| Gene symbol | Gene name | Known functions |
|---|---|---|
| FLI1 | Fli-1 Proto-Oncogene, ETS Transcription Factor | Encodes a transcription factor containing an ETS DNA-binding domain. |
| GNG5 | G Protein Subunit Gamma 5 | Facilitates glioblastoma cell growth, invasion, stemness and glycolysis via Wnt/$\beta$-catenin pathway. |
| FZD3 | Frizzled Class Receptor 3 | Controls early axon growth and guidance processes in central and peripheral fiber tracts. |
| GSK3B | Glycogen Synthase Kinase 3 Beta | Negative regulator of glucose homeostasis, Wnt signaling, transcription factors and microtubules. |
| CTNNB1 | Catenin Beta 1 | Blocks anoikis of malignant kidney and intestinal epithelial cells and promotes anchorage-independent growth. |
| RHOA | Ras Homolog Family Member A | Regulates platelet alpha-granule release during activation and aggregation. |
| FZD4 | Frizzled Class Receptor 4 | Critical for retinal vascularization; receptor for Wnt proteins and norrin (NDP). |
