# Supplementary material for: TransMarker: Unveiling dynamic network biomarkers in cancer progression through cross-state graph alignment and optimal transport
Source: PLoS Comput Biol. 2025 Nov 24;21(11):e1013743. doi: 10.1371/journal.pcbi.1013743 (PMC12668635; doi:10.1371/journal.pcbi.1013743)
Supplement: S2 Text — (PDF) [file pcbi.1013743.s014.pdf]

S2. Robustness of TransMarker to the selection and completeness of the prior knowledge network

To evaluate the robustness of TransMarker regarding the choice and completeness of the prior gene–gene interaction network, we performed additional experiments on the GAC dataset under two scenarios: (1) Alternative prior networks, where RegNetwork was replaced with HumanNet [26], InBioMap [27], and STRINGdb [28]; and (2) Edge removal perturbations, where 5%, 10%, 15%, and 20% of edges were randomly removed from RegNetwork to simulate incomplete prior knowledge.

The model was trained and evaluated under the same conditions to the main experiments. Across all scenarios, TransMarker maintained stable and high predictive performance (S7 Fig). Using HumanNet, InBioMap, and STRINGdb produced results comparable to RegNetwork, with AUROC values of 0.882, 0.868, and 0.912, respectively. Similarly, even after progressive edge removal, TransMarker retained AUROC values above 0.87, indicating limited sensitivity to missing connections.

In particular, ACC and AUPRC remained nearly unchanged with up to 10% edge removal, and only a slight decrease was observed when more than 15% of edges were removed. This stability suggests that TransMarker effectively utilizes both topological and learned embedding features, avoiding over-reliance on specific prior network structures.

References

[1] Wang R, Song S, Qin J, et al. Evolution of immune and stromal cell states and ecotypes during gastric adenocarcinoma progression. Cancer Cell. 2023;41(8):1407-1426.e9.

[2] Zhang P, Yang M, Zhang Y, et al. Dissecting the Single-Cell Transcriptome Network Underlying Gastric Premalignant Lesions and Early Gastric Cancer. Cell Rep. 2019;27(6):1934-1947.e5.

[3] Sathe A, Grimes SM, Lau BT, et al. Single-Cell Genomic Characterization Reveals the Cellular Reprogramming of the Gastric Tumor Microenvironment. Clin Cancer Res. 2020;26(11):2640-2653.

[4] Liu T, Zhao X, Lin Y, et al. Computational identification of preneoplastic cells displaying high stemness and risk of cancer progression. *Cancer Res.* 2022;82(14):2520–2537.

[5] Liu ZP, Wu C, Miao H, et al. RegNetwork: an integrated database of transcriptional and post-transcriptional regulatory networks in human and mouse. Database. 2015;2015:bav095.

[6] Dibaeinia P, Sinha S. SERGIO: A Single-Cell Expression Simulator Guided by Gene Regulatory Networks. Cell Syst. 2020;11(3):252-271.e11.

[7] Butler A, Hoffman P, Smibert P, et al. Integrating single-cell transcriptomic data across different conditions, technologies, and species. Nat Biotechnol. 2018;36(5):411-420.

[8] Wang D, Tian F, Wei D. A new centrality ranking method for multilayer networks. Journal of Computational Science. 2023;66:101924.

[9] Pan W, Ming H, Chang CK, et al. ElementRank: Ranking java software classes and packages using a multilayer complex network-based approach. IEEE Transactions on Software Engineering. 2019;47(10):2272–2295.

[10] De Domenico M, Solé-Ribalta A, Omodei E, et al. Ranking in interconnected multilayer networks reveals versatile nodes. Nature Communications. 2015;6(1):6868.

[11] Saha S, Bandyopadhyay S. Versatility-preserving multi-omics data analysis by ranking the nodes in multilayer network. In: 2020 IEEE 5th International Conference on Computing Communication and Automation (ICCCA). 2020;617–622.

[12] Wu M, He S, Zhang Y, et al. A tensor-based framework for studying eigenvector multicentrality in multilayer networks. Proceedings of the National Academy of Sciences. 2019;116(31):15407–15413.

[13] Brandes U. On variants of shortest-path betweenness centrality and their generic computation. Social Networks. 2008;30:136–145.

[14] Faghani MR, Nguyen UT. A study of XSS worm propagation and detection mechanisms in online social networks. IEEE Transactions on Information Forensics and Security. 2013;8:1815–1826.

[15] Freeman LC. Centrality in social networks conceptual clarification. Social Networks. 1978;1:215–239.

[16] Pal SK, Kundu S, Murthy CA. Centrality measures, upper bound, and influence maximization in large scale directed social networks. Fundamenta Informaticae. 2014;130:317–342.

[17] Opsahl T, Agneessens F, Skvoretz J. Node centrality in weighted networks: generalizing degree and shortest paths. Social Networks. 2010;32:245–251.

[18] Boldi P, Vigna S. Axioms for centrality. Internet Mathematics. 2014;10:222–262.

[19] Qi X, Fuller E, Wu Q, et al. Laplacian centrality: a new centrality measure for weighted networks. Information Sciences. 2012;194:240–253.

[20] Chen D, Lü L, Shang MS, et al. Identifying influential nodes in complex networks. Physica A. 2012;391:1777–1787.

[21] Lu L, Zhang YC, Yeung CH, et al. Leaders in social networks, the Delicious case. PLoS One. 2011;6:e21202.

[22] Joyce KE, Laurienti PJ, Burdette JH, et al. A new measure of centrality for brain networks. PLoS One. 2010;5:e12200.

[23] Dangalchev C. Residual closeness in networks. Physica A. 2006;365:556–564.

[24] Valente TW, Foreman RK. Integration and radiality: measuring the extent of an individual's connectedness and reachability in a network. Social Networks. 1998;20:89–105.

[25] Page L, Brin S, Motwani R, et al. The PageRank Citation Ranking: Bringing Order to the Web. Stanford InfoLab. 1999.

[26] Kim CY, Baek S, Cha J, Yang S, Kim E, Marcotte EM, Hart T, Lee I. HumanNet v3: an improved database of human gene networks for disease research. Nucleic Acids Research. 2022;50(D1):D632–D639.

[27] Lage K, Karlberg EO, Størling ZM, Olason PI, Pedersen AG, Rigina O, Hinsby AM, Tümer Z, Pociot F, Tommerup N, et al. A human phenome–interactome network of protein complexes implicated in genetic disorders. Nature Biotechnology. 2007;25(3):309–316.

[28] Szklarczyk D, Gable AL, Lyon D, Junge A, Wyder S, Huerta-Cepas J, Simonovic M, Doncheva NT, Morris JH, Bork P, et al. STRING v11: protein–protein association networks with increased coverage, supporting functional discovery in genome-wide experimental datasets. Nucleic Acids Research. 2019;47(D1):D607–D613.
