# Supplementary material for: TransMarker: Unveiling dynamic network biomarkers in cancer progression through cross-state graph alignment and optimal transport
Source: PLoS Comput Biol. 2025 Nov 24;21(11):e1013743. doi: 10.1371/journal.pcbi.1013743 (PMC12668635; doi:10.1371/journal.pcbi.1013743)
Supplement: S3 Text — (PDF) [file pcbi.1013743.s016.pdf]

S3. Robustness to the Entropic Regularization Parameter in the Gromov–Wasserstein Alignment

The Gromov–Wasserstein (GW) alignment step in *TransMarker* includes an entropic regularization term that controls the smoothness of the optimal transport plan. This parameter, denoted by $\mathcal{E}$, balances numerical stability and transport sparsity. We empirically set $\mathcal{E} = 1e-2$ as the default value, offering a good trade-off between convergence speed and biological interpretability. Specifically, smaller values ($\mathcal{E} < 1e-3$) resulted in highly sparse, unstable transport matrices, while larger values ($\mathcal{E} > 1e-1$) produced over-smoothed alignments that diluted biologically meaningful gene correspondences across states.

To assess the robustness of *TransMarker* to this parameter, we performed a sensitivity analysis using the GAC dataset. The parameter $\mathcal{E}$ was varied over $\{1e-3, 5e-3, 1e-2, 5e-2, 1e-1\}$ while all other settings fixed. For each value, we recomputed the cross-state alignments and recalculated the Dynamic Network Index (DNI) scores for all genes.

The results (S8A Fig) show that classification metrics remain acceptable across the parameter range. The Spearman rank correlation between the default setting ($\mathcal{E} = 1e-2$) and other values exceeded 0.92 (S8B Fig), indicating that biomarker prioritization is not sensitive to the specific regularization choice. Moreover, the average rank deviation among the top 20 biomarkers was below two positions, and their DNI scores showed negligible variation (S8C Fig). These findings confirm that the TransMarker framework maintains consistent performance and reliable biomarker prioritization over a reasonable range of entropic regularization strengths.
