# Supplementary material for: TransMarker: Unveiling dynamic network biomarkers in cancer progression through cross-state graph alignment and optimal transport
Source: PLoS Comput Biol. 2025 Nov 24;21(11):e1013743. doi: 10.1371/journal.pcbi.1013743 (PMC12668635; doi:10.1371/journal.pcbi.1013743)
Supplement: Attachment — Submitted filename: report.pdf [file pcbi.1013743.s018.pdf]

Review Report for PCOMPBIOL-D-25-01660

General Comments

This paper introduces “TransMarker”, a novel and promising computational framework for identifying dynamic network biomarkers (DNBs) in cancer progression. The authors leverage a sophisticated combination of graph attention networks (GATs) and optimal transport theory to quantify changes in the structural roles of genes across different disease states, defined from single-cell RNA-seq data. The method is validated using both simulated and real-world cancer datasets, where it demonstrates superior performance over existing methods.

The manuscript is well-written, and the proposed method represents a significant contribution to the field of computational biology. The core idea of identifying switcher genes based on changes in their network embedding is powerful. I recommend this paper for publication, provided that the authors address the following points.

Major Concerns and Suggestions for Improvement

I have a few major suggestions for the authors to consider. Addressing these points would improve the clarity of the paper.

1. **Applicability to Pseudo-time Ordered Data**
   The current study demonstrates the power of TransMarker on data with discrete, predefined states (e.g., normal vs. tumor). However, a very common use case for scRNA-seq is pseudo-time analysis, where cells from a single snapshot are ordered along a continuous trajectory of differentiation or disease progression. The TransMarker framework could, in principle, be applied to such data by dividing the pseudo-time continuum into discrete states (e.g., early, middle, late). This would dramatically broaden the applicability of the method. I strongly suggest that the authors add a discussion on this potential application in the Discussion section. They should discuss the opportunities (e.g., finding key drivers of state transitions) as well as the potential challenges (e.g., sensitivity to the pseudo-time inference method or the way states are segmented). If feasible, a validation on a public dataset with a well-established pseudo-time trajectory would make the paper stronger.

2. **Discussion on Scalability and Computational Cost**
   The proposed method combines several computationally intensive steps (GATs, optimal transport). It would be very helpful for readers and potential users if the authors could add

a brief discussion on the computational cost and scalability of TransMarker. For example, how does the computation time scale with the number of cells, genes, or disease states?

3. **Robustness to the Regularization Parameter of the Gromov–Wasserstein Distance**
One of the key steps in the proposed methodology is the computation of the Gromov–Wasserstein distance, which often involves an entropic regularization term controlled by a parameter. The choice of this regularization parameter can have a significant impact on the resulting optimal transport plan and, consequently, on the Dynamic Network Index (DNI) scores. It would greatly enhance the paper's rigor if the authors could elaborate on this aspect. Could they clarify how the value for this regularization parameter was selected? Furthermore, a sensitivity analysis demonstrating that the key findings, particularly the ranking of the top DNBs, are robust and stable across a reasonable range of this parameter's values would provide stronger confidence in the results and show that they are not artifacts of a specific parameter choice.

4. **Robustness to the Prior Knowledge Network**
The method relies on a pre-existing gene-gene interaction network. The quality and completeness of this prior network can vary and may influence the results. I suggest the authors discuss the potential impact of using different prior networks. A brief analysis of the method's robustness to noise (e.g., by removing a certain percentage of edges from the prior network) would strengthen the claims of the paper's robustness.

Minor Points

Finally, here are a few minor points and suggested corrections to improve the manuscript's readability.

- In the Abstract: "...biomarkers that **reflecting** dynamic changes..." should be "...biomarkers that **reflect** dynamic changes...".
- In the Abstract: Consider using hyphenated compound adjectives for clarity, e.g., "cross-state" and "multi-state". Please ensure consistency throughout the manuscript.
- Please perform a final proofread of the manuscript for minor typos and grammatical errors.
