# Supplementary material for: TransMarker: Unveiling dynamic network biomarkers in cancer progression through cross-state graph alignment and optimal transport
Source: PLoS Comput Biol. 2025 Nov 24;21(11):e1013743. doi: 10.1371/journal.pcbi.1013743 (PMC12668635; doi:10.1371/journal.pcbi.1013743)
Supplement: Attachment — Submitted filename: 20251023---Response-to-Decision Letter.docx [file pcbi.1013743.s019.docx]

**Manuscript ID: PCOMPBIOL-D-25-01660**

**Title:** TransMarker: Unveiling dynamic network biomarkers in cancer progression through cross-state graph alignment and optimal transport

Dear Professor Shi,

Thank you very much for granting us the opportunity to revise our manuscript titled “TransMarker: Unveiling dynamic network biomarkers in cancer progression through cross-state graph alignment and optimal transport” (PCOMPBIOL-D-25-01660). We are deeply grateful to all the three reviewers for their positive comments and insightful feedback, which have significantly improved our paper. We have diligently revised the manuscript in accordance with you and the reviewers’ constructive comments and suggestions, addressing each point individually. Furthermore, we have incorporated the comprehensive revisions outlined in your decision letter. Below is a brief summary of the main revisions:

1. Based on the reviewers’ feedback, we have made appropriate adjustments to the main text and supplementary materials. Changes have been highlighted in red font for easy reference in the revised manuscript.
2. Figure 1 has been modified to explicitly illustrate the step of Gromov–Wasserstein optimal transport, clarifying its role in the framework.
3. The optimization problem formulation now appears in a standard mathematical form to improve clarity and reproducibility.
4. We ensured consistent and sufficiently large font sizes (not smaller than 8) across all figures for better readability.
5. The naming conventions for all compared methods have been standardized across the text, tables, figure panels, and captions.
6. The “Comparison with related methods” section has been expanded to include concise explanations of each baseline method for clearer contextualization.
7. We have thoroughly proofread the entire manuscript to correct minor typographical and grammatical errors.
8. All compound adjectives have been revised for consistent hyphenation, adopting “cross-state” and “multi-state” throughout the manuscript.
9. We have expanded the Introduction to incorporate recent advances in optimal transport–based frameworks (TIGON, TrajectoryNet, DeepRUOT, stVCR) and Dynamic Network Biomarker–related methods (RSIT, stPCA, sPGGM, SCNE), as suggested by the reviewers, to provide broader theoretical context and highlight the relevance of our study within the latest computational developments.
10. We have added a new paragraph in the Discussion section addressing the applicability of TransMarker to pseudo-time ordered scRNA-seq data, discussing how the framework could be adapted for continuous trajectories, potential advantages in identifying transition regulators, and associated methodological challenges.
11. We have expanded the Discussion to include an analysis of computational cost and scalability, clarifying the complexity of the GAT and GWOT modules, their scaling behavior, and the framework’s efficient parallelization across disease states for large datasets.
12. We have added a new analysis and discussion in S3 Text (Supplementary Files), explaining how the entropic regularization parameter in the Gromov–Wasserstein alignment was selected and demonstrating the robustness of the framework to its variation.
13. We have conducted additional experiments (S7 Fig) using alternative prior network resources and by randomly removing up to 20% of edges from the original network to evaluate robustness to network incompleteness.
14. We have completed and verified the CRediT author contribution statements for all co-authors to ensure accuracy and compliance with journal requirements.
15. All figure files have been converted to .eps format in accordance with the journal’s submission guidelines.
16. A comprehensive list of legends for all Supporting Information files has been added after the References section, as requested.

Please find enclosed our detailed, point-by-point responses to the reviewers’ comments. We believe these revisions comprehensively address the reviewers’ feedback and further improve the overall quality and readability of the manuscript. We eagerly anticipate any further feedback from you and the reviewers.

Thank you once again for your continued support of our work.

Sincerely,

Zhi-Ping Liu, PhD, Professor

Director of Institute of Biomedical Engineering

Shandong University, Jinan, Shandong Province, China

[zpliu@sdu.edu.cn](mailto:zpliu@sdu.edu.cn)

**Reviewer #1**

***Comments to the Author:*** In this manuscript, the authors present a novel computational framework TransMarker for identifying dynamic biomarkers of cancer progression using single-cell RNA-seq data. The central idea is that cancer evolution involves not only changes in expression levels but also rewiring of gene regulatory networks across disease states. The framework integrates prior regulatory knowledge with state-specific gene expression to construct GRNs, where Graph Attention Networks (GATs) are used to learn contextual embeddings for each state, and Gromov-Wasserstein optimal transport quantifies structural shifts in gene roles across states. The significant changed genes are then used for disease state classification as DNBs. The authors validate TransMarker through both synthetic simulations and real-world datasets such as GAC and ESCC with systematic benchmarking and rigorous ablation studies, as well biological interpretability analysis.

Overall, this is a strong submission and solid work, and the manuscript is very well-written. I suggest the acceptance of manuscript by PlosCB.

*Q1. I have only one minor suggestion for authors to consider if that’s appropriate: Recently, there are several dynamical OT based methods to analyze scRNA-seq data (e.g. TIGON, NMI 2024; TrajectoryNet,ICML 2020; DeepRUOT, ICLR 2025; srVCR, Biorxiv 2024), and inference of GRN is also possible from these approaches. The authors might discuss these related works in the intro/discussion of the manuscript to further enhance the interests for general audience.*

**A1:** We sincerely thank the reviewer for this insightful suggestion. We fully agree that recent dynamical optimal transport (OT)-based methods, such as TIGON [1], TrajectoryNet [2], DeepRUOT [3], and stVCR [4], are exciting developments in modeling dynamic cellular processes using single-cell RNA-seq data. Following the reviewer’s recommendation, we have added a short discussion in the Introduction section to highlight these works and emphasize the growing role of OT frameworks in understanding temporal regulatory mechanisms and gene regulatory network inference.

“Recently, several dynamical optimal transport (OT)–based frameworks have emerged for modeling temporal cell-state transitions and inferring gene regulatory dynamics from single-cell transcriptomic data. Notable examples include TrajectoryNet [27], which models continuous developmental trajectories using neural OT; TIGON [28], which integrates OT with graph neural networks to uncover temporally evolving regulatory interactions; DeepRUOT [29], which extends regularized unbalanced OT for robust cross-time gene expression alignment; and stVCR [30], which employs stochastic regularization to improve velocity-consistent reconstruction of single-cell trajectories. These approaches collectively highlight the power of OT for capturing dynamic molecular processes and inferring regulatory relationships from continuous transitions. Inspired by these developments, our work extends the OT principle to cross-state network alignment in cancer, focusing on identifying dynamic network biomarkers that reflect regulatory rewiring across discrete pathological states rather than continuous cell transitions.”

These additions help situate our study within the broader context of dynamic OT-based biological modeling and clarify how TransMarker extends OT principles to disease-state network alignment and dynamic biomarker discovery.

We thank the reviewer once again and hope that this response and the provided files meet your expectations.

**Reviewer #2**

***Comments to the Author:*** The manuscript presents TransMarker, a multilayer network framework that integrates prior gene–gene interactions, state-specific single-cell expression, and Gromov–Wasserstein optimal transport to identify dynamic network biomarkers across cancer progression. The method is well motivated, the experiments are thorough, and the results convincingly demonstrate superiority over existing multilayer ranking approaches. The paper is generally well written and of interest to the computational biology community. I listed here some comments and presentation suggestions to further improve clarity and reproducibility:

*Q1. Framework Figure (Figure 1): In panel 1-E (Graph Alignment step), the role of optimal transport is central but not explicitly illustrated. Please modify the figure to depict the Gromov–Wasserstein optimal transport step so that the reader can visually follow this critical component.*

**A1:** We are deeply appreciating your valuable insights. We totally agree with your comment regarding the need to more clearly illustrate the role of optimal transport in the Graph Alignment step. Following your suggestion, we have revised the framework figure (Fig 1) to explicitly depict the Gromov–Wasserstein (GW) optimal transport process. In the updated panel fig 1-E, we now highlight the GW mapping between the two networks and include a concise label showing the optimal transport flow, allowing readers to visually track how the alignment is achieved. Thank you again for your valuable suggestion.

*Q2. Optimization Problem Formulation: After line 591, where the Gromov–Wasserstein distance is introduced, please provide the explicit standard mathematical form of the optimization problem. This will help readers reproduce the method without consulting external sources.*

**A2:** We sincerely appreciate the reviewer’s thoughtful comment. we have added the explicit mathematical formulation of the Gromov–Wasserstein optimization problem in the revised manuscript (after line 591) to facilitate reproducibility. The formulation now appears in the standard form as follows:

This modification provides a clear and self-contained presentation of the optimization problem and aligns with the standard formulation used in optimal transport literature. We hope this additional information addresses the reviewer’s concerns regarding the clarity and reproducibility of our method.

*Q3.* *Figure Font Consistency: Ensure consistent and sufficiently large font sizes across all figures. In particular, Figures 2, 3, 4, and 6 contain axis labels and legends that are noticeably smaller than in other panels, making them difficult to read.*

**A3:** Your comment is greatly appreciated. We sincerely thank the reviewer for highlighting the issue of font consistency across figures. In the revised version of the manuscript, we have thoroughly reviewed and updated all figures to ensure uniform and clearly legible font sizes for axis labels, legends, and annotations. Specifically, Figures 2, 3, 4, and 6 have been redrawn with enlarged and harmonized fonts, ensuring readability and consistency across all visual elements.

Thank you again for pointing this out. Your valuable feedback has greatly improved the visual clarity and overall presentation quality of our figures.

*Q4. Clarification of Comparative Methods: In the “Comparison with highly related methods” section, please expand the discussion of the five multilayer node-ranking methods used for benchmarking. For each, briefly explain which aspects of node importance across multilayer topologies they capture. This contextualization will help readers understand why these particular methods were chosen and how their design principles differ from TransMarker’s integration of regulatory rewiring and optimal transport.*

**A4:** We sincerely appreciate the reviewer’s insightful comment. In response, we have expanded the “Comparison with highly related methods” section to include a concise explanation of each of the five multilayer node-ranking methods used for benchmarking. Specifically, we now clarify the key principles and distinctive features that each method captures regarding node importance across multilayer topologies.

In the revised manuscript, we explain that:

**Multilayer Entropy** quantifies node importance using structural information entropy from intra- and inter-layer connections, emphasizing nodes that bridge multiple layers.

**ElementRank** extends PageRank to multilayer networks by integrating layer-specific influence through weighted aggregation, reflecting both local and global importance.

**Versatility Centrality** measures a node’s eigenvector centrality across layers using a tensor-based framework, highlighting nodes with consistently high influence in the multilayer system.

**Versatility Degree Centrality** aggregates degree information across layers to identify nodes strongly connected and active in multiple layers, showing consistent cross-layer significance.

**Eigenvector Multicentrality** generalizes eigenvector centrality via tensor decomposition to jointly capture within-layer and between-layer influence propagation.

These clarifications highlight the unique design focus of each baseline method and show how TransMarker differs conceptually by incorporating regulatory rewiring and optimal transport to model dynamic state transitions rather than static multilayer centrality.

We have added this summary paragraph to the “Comparison with highly related methods” section, and the detailed mathematical and performance descriptions are still available in Supplementary S1 Text.

“Each of these multilayer node-ranking methods captures distinct aspects of node importance across complex multilayer topologies. Specifically, Multilayer Entropy evaluates nodes based on structural information entropy, emphasizing those bridging different layers. ElementRank extends the PageRank algorithm by weighting layer-specific importance to reflect both local and global influence. Versatility Centrality employs a tensor-based approach to aggregate eigenvector centrality across layers, highlighting nodes consistently influential throughout the network. Versatility Degree Centrality integrates degree information from multiple layers to identify nodes with stable cross-layer connectivity. Eigenvector Multicentrality generalizes eigenvector centrality using a tensor model that simultaneously accounts for intra-layer and inter-layer propagation effects. These methods offer complementary perspectives on multilayer structure; however, unlike TransMarker, they do not explicitly capture dynamic regulatory rewiring or state-specific transitions using optimal transport principles, which are central to our framework.”

Thank you again for your constructive feedback, which has significantly improved the clarity and interpretability of our benchmarking discussion.

*Q5.* *Typographical consistency: There are several instances of inconsistent naming of the compared methods. For example, the same method appears with different capitalization and occasional extra characters (e.g., a dash or underscore) in Table 2 versus Figure 6A. Please standardize the naming convention for all methods across the main text, tables, figure panels, and captions. Ensure that each method’s name is written exactly the same where it is introduced, discussed in the Results, and labeled in figures. In particular, correct the mismatch between Table 2 and Figure 6A. Adopting a uniform style will improve clarity.*

**A5:** We sincerely appreciate the reviewer’s insightful comment. We fully agree that maintaining consistent typographical conventions is essential for clarity and readability. Accordingly, we have carefully revised the entire manuscript, including the main text, tables, figure panels, and captions, to ensure a uniform naming style for all compared methods.

Specifically, we have standardized the method names to appear consistently as Multilayer Entropy, ElementRank, Versatility Centrality, Versatility Degree Centrality, and Eigenvector Multicentrality throughout the manuscript.

Thank you again for your valuable and constructive feedback, which has helped us improve the consistency and presentation quality of our paper.

*Q6. Some most recent computational methods for identifying the critical states based on the dynamic network biomarker (DNB) or its modifications are missing. I suggest the following recent and relevant literatures should be addressed:*

*- Earthquake alerting based on spatial geodetic data by spatiotemporal information transformation learning. Proceedings of the National Academy of Sciences, USA, 2023, 120(37):e2302275120.*

*- Ultralow-dimensionality reduction for identifying critical transitions by spatial-temporal PCA. Advanced Science, 2025, 9:2408173.*

*- sPGGM: a sample-perturbed Gaussian graphical model for identifying pre-disease stages and signaling molecules of disease progression. National Science Review, 2025: nwaf189.*

*- Uncovering the pre-deterioration state during disease progression based on sample-specific causality network entropy (SCNE). Research, 2024, 7:0368.*

**A5:** We sincerely appreciate the reviewer’s thoughtful suggestion and for drawing our attention to these important recent studies. In response, we have expanded the Introduction section to include a concise overview of these dynamic network biomarker–related frameworks, which further contextualize the motivation and relevance of our work.

Specifically, we now discuss the latest advances in DNB-inspired methodologies that explore early-warning and critical-transition detection using dynamic and spatiotemporal models. These include the spatiotemporal information transformation learning approach for large-scale systems such as earthquakes [5], the spatial–temporal PCA framework for identifying critical transitions in high-dimensional data [6], the sPGGM model for revealing pre-disease stages through perturbed network inference [7], and the SCNE method for detecting pre-deterioration states via causality-based entropy analysis [8].

These works collectively reinforce the conceptual foundation of our study by underscoring the increasing importance of DNB-based approaches across various domains. The revised manuscript incorporates these additions, which we have marked in red font for clarity.

“Recent studies have extended Dynamic Network Biomarker research by devising advanced frameworks for detecting early-warning and critical-transition signals in complex systems. Methods such as spatiotemporal information transformation learning (RSIT) [23] and spatial–temporal PCA (stPCA) [24] identify system instability and tipping points from high-dimensional data. In disease modeling, the sample-perturbed Gaussian graphical model (sPGGM) [25] and sample-specific causality network entropy (SCNE) [26] uncover pre-disease or pre-deterioration states via individualized network analysis. These approaches emphasize the growing significance of DNB-inspired methods for detecting critical transitions and inspire the design of our framework.”

We thank the reviewer once again and hope that this response and the provided files meet your expectations.

**Reviewer #3**

***Comments to the Author:*** This paper introduces “TransMarker”, a novel and promising computational framework for identifying dynamic network biomarkers (DNBs) in cancer progression. The authors leverage a sophisticated combination of graph attention networks (GATs) and optimal transport theory to quantify changes in the structural roles of genes across different disease states, deﬁned from single-cell RNA-seq data. The method is validated using both simulated and real-world cancer datasets, where it demonstrates superior performance over existing methods.

The manuscript is well-written, and the proposed method represents a signiﬁcant contribu-tion to the ﬁeld of computational biology. The core idea of identifying switcher genes based on changes in their network embedding is powerful. I recommend this paper for publication, provided that the authors address the following points.

***Major Concerns and Suggestions for Improvement***

***I have a few major suggestions for the authors to consider. Addressing these points would improve the clarity of the paper.***

*Q1.* ***Applicability to Pseudo-time Ordered Data***

*The current study demonstrates the power of TransMarker on data with discrete, pre-deﬁned states (e.g., normal vs. tumor). However, a very common use case for scRNA-seq is pseudo-time analysis, where cells from a single snapshot are ordered along a continuous trajectory of differentiation or disease progression. The TransMarker framework could, in principle, be applied to such data by dividing the pseudo-time continuum into discrete states (e.g., early, middle, late). This would dramatically broaden the applicability of the method. I strongly suggest that the authors add a discussion on this potential application in the Discussion section. They should discuss the opportunities (e.g., ﬁnding key drivers of state transitions) as well as the potential challenges (e.g., sensitivity to the pseudo-time inference method or the way states are segmented). If feasible, a validation on a public dataset with a well-established pseudo-time trajectory would make the paper stronger.*

**A1:** We sincerely thank the reviewer for this insightful suggestion. We fully agree that adapting TransMarker to pseudo-time ordered scRNA-seq data is an exciting avenue for future work. In response, we have added a dedicated paragraph in the Discussion section outlining how the framework could be modified for continuous trajectories by dividing the pseudo-time continuum into discrete intervals (e.g., early, middle, late) to track dynamic network reconfiguration during cellular differentiation or disease progression. We also address the potential benefits, such as identifying key regulators of state transitions, and challenges, including sensitivity to pseudo-time inference accuracy and state segmentation strategies. This addition underscores the broader applicability of TransMarker to dynamic single-cell systems beyond predefined disease states.

“Recent single-cell studies frequently model biological processes along continuous pseudo-time trajectories rather than discrete disease states. TransMarker could be adapted to such data by discretizing the pseudo-time continuum into ordered segments (e.g., early, middle, and late stages) and constructing corresponding regulatory networks for each segment. This would enable the framework to capture dynamic rewiring patterns and identify key transition regulators driving differentiation or disease progression along pseudo-temporal trajectories. Such an adaptation could provide valuable insights into the timing and sequence of molecular events underlying critical transitions. However, challenges include potential sensitivity to the pseudo-time inference method, variability in trajectory reconstruction across algorithms, and the need for principled segmentation strategies to balance temporal resolution and robustness. Future work will evaluate TransMarker on benchmark pseudo-time datasets to systematically assess its performance in reconstructing and prioritizing dynamic biomarkers in continuous single-cell processes.”

We sincerely appreciate the reviewer’s constructive feedback, which helped us clarify this potential extension and its implications for TransMarker’s broader applicability.

*Q2.* ***Discussion on Scalability and Computational Cost***

*The proposed method combines several computationally intensive steps (GATs, optimal transport). It would be very helpful for readers and potential users if the authors could add a brief discussion on the computational cost and scalability of TransMarker. For example, how does the computation time scale with the number of cells, genes, or disease states?*

**A2:** We thank the reviewer for this valuable suggestion. While the formal time complexity of TransMarker is already detailed in Section “*Complexity Analysis*”, we have now added a complementary paragraph in the Discussion section that provides an intuitive interpretation of computational scalability in practical settings.

Specifically, we clarify how the main components of TransMarker scale with the number of genes, edges, and disease states. The graph attention network (GAT) module scales approximately linearly with the number of edges in each state-specific network, whereas the Gromov–Wasserstein optimal transport (GWOT) alignment step scales quadratically with the number of nodes but can be substantially reduced through entropic regularization and mini-batch optimization. Importantly, since TransMarker operates on state-level gene networks (rather than per-cell graphs), the overall computation remains tractable even for large transcriptomic datasets.

In practice, we evaluated TransMarker on the GAC dataset, which contains five disease states (NAT, CAG, IM, PGAC, and Metastasis) with approximately 10,000 genes per state.

The state-specific GAT embedding for all five networks required about 1.5 hours on a single NVIDIA Quadro RTX 6000 GPU (24 GB VRAM), with a peak GPU memory usage of approximately 13 GB.

The subsequent GWOT-based alignment across four consecutive state transitions required an additional 1 hour on the same hardware.

Overall, the complete TransMarker pipeline for the GAC dataset finished in under 3 hours, with total memory consumption remaining below 14 GB, while the workstation had 251 GB of system memory available.

Runtime scales approximately linearly with the number of disease states (since each state can be processed in parallel) and roughly quadratically with the number of genes, consistent with the theoretical complexity analysis. These empirical results confirm that TransMarker is computationally efficient and practically scalable for large-scale, state-resolved transcriptomic studies.

The following paragraph has been added to the Discussion section:

“From a computational perspective, TransMarker combines topological learning, GAT-based embedding, and Gromov–Wasserstein optimal transport alignment across disease states. The GAT component scales approximately linearly with the number of edges, while the GWOT step scales roughly with the square of the number of nodes but can be efficiently approximated through entropic regularization and mini-batch computation. When applied to the GAC dataset (five disease states, ~10,000 genes each), the complete training and alignment process took about 3 hours on a single NVIDIA Quadro RTX 6000 GPU (24 GB VRAM), with a peak memory footprint of ~13 GB. Since TransMarker operates on state-level networks and supports parallel processing across states, it remains computationally feasible and scalable for large multi-state disease analyses.”

We sincerely thank the reviewer for prompting us to include this practical discussion, which clarifies the scalability and computational performance of our framework beyond the formal complexity analysis.

*Q3.* ***Robustness to the Regularization Parameter of the Gromov–Wasserstein Distance***

*One of the key steps in the proposed methodology is the computation of the Gromov–Wasserstein distance, which often involves an entropic regularization term controlled by a parameter. The choice of this regularization parameter can have a signiﬁcant impact on the resulting optimal transport plan and, consequently, on the Dynamic Network Index (DNI) scores. It would greatly enhance the paper’s rigor if the authors could elaborate on this aspect. Could they clarify how the value for this regularization parameter was selected? Furthermore, a sensitivity analysis demonstrating that the key ﬁndings, particularly the ranking of the top DNBs, are robust and stable across a reasonable range of this parameter’s values would provide stronger conﬁdence in the results and show that they are not artifacts of a speciﬁc parameter choice.*

**A3:** We sincerely thank the reviewer for this insightful suggestion regarding the entropic regularization parameter in the Gromov–Wasserstein (GW) alignment. We have now included a detailed analysis and discussion in S3 Text (Supplementary files) to clarify how this parameter was selected and to evaluate the robustness of our framework to its variation.

In our implementation, the entropic regularization term ($\mathcal{E}$) controls the smoothness of the optimal transport plan, balancing numerical stability and transport sparsity. We empirically set $\mathcal{E}=1e-2$ as the default value, as it offered an optimal trade-off between convergence efficiency and biological interpretability. To rigorously assess robustness, we performed a sensitivity analysis using the GAC dataset, varying $\mathcal{E}$ across {1e−3, 5e−3, 1e−2, 5e−2, 1e−1} while keeping all other parameters fixed. As shown in S8 Figure the results demonstrate that:

(i) Classification metrics (ACC, AUROC, AUPRC) remained consistently high across the entire parameter range;
(ii) The Spearman rank correlation of gene prioritizations relative to the default setting ($\mathcal{E}=1e-2$) exceeded 0.92; and
(iii) The mean rank deviation among the top 20 biomarkers was below two positions, with negligible variation in their DNI scores.

These findings confirm that the TransMarker framework is robust to the choice of entropic regularization, maintaining consistent performance and stable biomarker rankings across a reasonable range of parameter values.

“**S3 Text. Robustness to the Entropic Regularization Parameter in the Gromov–Wasserstein Alignment**

The Gromov–Wasserstein (GW) alignment step in TransMarker includes an entropic regularization term that controls the smoothness of the optimal transport plan. This parameter, denoted by 𝓔, balances numerical stability and transport sparsity. We empirically set $\mathcal{E}=1e-2$ as the default value, offering a good trade-off between convergence speed and biological interpretability. Specifically, smaller values $\mathcal{(E<}1e-3)$ resulted in highly sparse, unstable transport matrices, while larger values $\mathcal{(E>}1e-1)$ produced over-smoothed alignments that diluted biologically meaningful gene correspondences across states.

To assess the robustness of TransMarker to this parameter, we performed a sensitivity analysis using the GAC dataset. The parameter 𝓔 was varied over $\{1e-3, 5e-3,1e-2,5e-2,1e-1\}$ while all other settings fixed. For each value, we recomputed the cross-state alignments and recalculated the Dynamic Network Index (DNI) scores for all genes.

The results (S8A Fig) show that classification metrics remain acceptable across the parameter range. The Spearman rank correlation between the default setting ($\mathcal{E}=1e-2$) and other values exceeded 0.92 (S8B Fig), indicating that biomarker prioritization is not sensitive to the specific regularization choice. Moreover, the average rank deviation among the top 20 biomarkers was below two positions, and their DNI scores showed negligible variation (S8C Fig). These findings confirm that the TransMarker framework maintains consistent performance and reliable biomarker prioritization over a reasonable range of entropic regularization strengths.”

**Fig S8. Robustness analysis of the entropic regularization parameter (ε) in the Gromov–Wasserstein alignment step of TransMarker.** (A) Classification performance (ACC, AUROC, and AUPRC) across ε ∈ {1e−3, 5e−3, 1e−2, 5e−2, 1e−1}. (B) Spearman rank correlation of gene prioritizations compared to the default ε = 1e−2. (C) Mean rank deviation of the top 20 biomarkers across different ε values.

We have mentioned this new section of the Supplementary Information in the main text as follows: “Further robustness analyses are provided in the Supplementary Information. Specifically, S2 Text and S7 Fig investigate the robustness of TransMarker to the choice and completeness of the prior knowledge network, while S3 Text and S8 Fig assess the impact of the entropic regularization parameter$\left( \mathcal{E} \right)$ in the Gromov–Wasserstein alignment step.”

Thank you again for this valuable feedback, which helped us enhance the methodological rigor and interpretability of our approach.

*Q4.* ***Robustness to the Prior Knowledge Network***

*The method relies on a pre-existing gene-gene interaction network. The quality and completeness of this prior network can vary and may inﬂuence the results. I suggest the authors discuss the potential impact of using different prior networks. A brief analysis of the method’s robustness to noise (e.g., by removing a certain percentage of edges from the prior network) would strengthen the claims of the paper’s robustness.*

**A4:** We thank the reviewer for this insightful suggestion. To evaluate the robustness of TransMarker to the choice and completeness of the prior interaction network, we conducted additional experiments on the GAC dataset using three alternative gene–gene interaction resources (HumanNet, InBio Map, and STRINGdb) in place of RegNetwork. Furthermore, to simulate incomplete or noisy prior networks, we randomly removed 5%, 10%, 15%, and 20% of edges from the original RegNetwork.

As shown in Supplementary Figure S7, TransMarker achieved consistently high performance across all network variants. The results using HumanNet (ACC: 0.860 ± 0.014, AUROC: 0.882 ± 0.013, AUPRC: 0.866 ± 0.015), InBio Map (0.840 ± 0.039, 0.868 ± 0.034, 0.850 ± 0.034), and STRINGdb (0.887 ± 0.031, 0.912 ± 0.027, 0.881 ± 0.028) were comparable to those obtained with RegNetwork (0.876 ± 0.010, 0.923 ± 0.011, 0.887 ± 0.012). When random edge removals were introduced, TransMarker remained robust, with only marginal performance degradation even at 20% edge removal (–5%: 0.901 ± 0.020, 0.916 ± 0.020, 0.904 ± 0.020; –10%: 0.895 ± 0.026, 0.911 ± 0.025, 0.896 ± 0.026; –15%: 0.851 ± 0.027, 0.875 ± 0.029, 0.888 ± 0.019; –20%: 0.852 ± 0.027, 0.870 ± 0.024, 0.859 ± 0.033). These results confirm that TransMarker’s performance is stable across different prior network sources and resilient to partial network incompleteness, highlighting its robustness and general applicability.

“**S2 Text. Robustness of TransMarker to the selection and completeness of the prior knowledge network**

To evaluate the robustness of TransMarker regarding the choice and completeness of the prior gene–gene interaction network, we performed additional experiments on the GAC dataset under two scenarios: (1) Alternative prior networks, where RegNetwork was replaced with HumanNet [26], InBioMap [27], and STRINGdb [28]; and (2) Edge removal perturbations, where 5%, 10%, 15%, and 20% of edges were randomly removed from RegNetwork to simulate incomplete prior knowledge.

The model was trained and evaluated under the same conditions to the main experiments. Across all scenarios, TransMarker maintained stable and high predictive performance (S7 Fig). Using HumanNet, InBioMap, and STRINGdb produced results comparable to RegNetwork, with AUROC values of 0.882, 0.868, and 0.912, respectively. Similarly, even after progressive edge removal, TransMarker retained AUROC values above 0.87, indicating limited sensitivity to missing connections.

In particular, ACC and AUPRC remained nearly unchanged with up to 10% edge removal, and only a slight decrease was observed when more than 15% of edges were removed. This stability suggests that TransMarker effectively utilizes both topological and learned embedding features, avoiding over-reliance on specific prior network structures.”

**Fig S7. Robustness of TransMarker to the selection and completeness of the prior knowledge network.** Performance comparison of TransMarker when using different prior interaction networks (HumanNet, InBioMap, STRINGdb) and under progressive random edge removal (–5%, –10%, –15%, and –20%) from the original RegNetwork prior. Bars represent mean ± standard deviation across multiple runs for Accuracy (ACC), AUROC, and AUPRC.

We have referenced this new section of the Supplementary Information in the main text as follows: “Further robustness analyses are provided in the Supplementary Information. Specifically, S2 Text and S7 Fig examine the robustness of TransMarker to the selection and completeness of the prior knowledge network, while S3 Text and S8 Fig analyze the influence of the entropic regularization parameter$\left( \mathcal{E} \right)$ in the Gromov–Wasserstein alignment step.”

We sincerely thank the reviewer for this thoughtful comment, which prompted us to conduct additional robustness analyses. These experiments provided valuable confirmation that TransMarker maintains consistent performance across diverse prior networks and under varying levels of network incompleteness, further strengthening the reliability and generality of our framework.

***Minor Points***

***Finally, here are a few minor points and suggested corrections to improve the manuscript’s readability.***

*Q5. In the Abstract: ”...biomarkers that* ***reﬂecting*** *dynamic changes...” should be ”...biomarkers that* ***reﬂect*** *dynamic changes...”.*

**A5:** Thank you for your valuable feedback. In response, we have corrected this grammatical error in the Abstract by replacing “biomarkers that reﬂecting dynamic changes” with “biomarkers that reﬂect dynamic changes.” We appreciate the reviewer’s careful attention to detail, which has helped improve the accuracy and readability of the manuscript.

*Q6.* In the Abstract: Consider using hyphenated compound adjectives for clarity, e.g., “cross-state” and “multi-state”. Please ensure consistency throughout the manuscript.

**A6:** We sincerely appreciate the reviewer’s valuable suggestion. Following your advice, we have carefully reviewed the entire manuscript and revised all instances of compound adjectives to ensure consistent and grammatically correct usage. Specifically, we have adopted the hyphenated forms “cross-state”, “multi-state” and “Gromov–Wasserstein” throughout the Abstract and the full text for clarity and uniformity.

Thank you again for your constructive feedback, which has improved the stylistic consistency and readability of the manuscript.

*Q7.* Please perform a final proofread of the manuscript for minor typos and grammatical

errors.

**A7:** We sincerely appreciate the reviewer’s thoughtful reminder. In response, we have carefully re-read and thoroughly proofread the entire manuscript to correct minor typographical and grammatical errors. All necessary revisions have been made to ensure linguistic accuracy, consistency, and overall clarity.

Thank you again for your helpful suggestion, which has contributed to improving the readability and presentation quality of our paper.

We thank the reviewer once again and hope that this response and the provided files meet your expectations.

# References

# [1] Tong A, Huang J, Wolf G, Van Dijk D, Krishnaswamy S. TrajectoryNet: A dynamic optimal transport network for modeling cellular dynamics. In: International Conference on Machine Learning. 2020;9526–9536.

# [2] Sha Y, Qiu Y, Zhou P, Nie Q. Reconstructing growth and dynamic trajectories from single-cell transcriptomics data. Nat Mach Intell. 2024;6(1):25–39.

# [3] Zhang Z, Li T, Zhou P. Learning stochastic dynamics from snapshots through regularized unbalanced optimal transport. In: The Thirteenth International Conference on Learning Representations. 2025.

# [4] Peng Q, Zhou P, Li T. stVCR: Reconstructing spatio-temporal dynamics of cell development using optimal transport. bioRxiv. 2024.

# [5] Tong Y, Hong R, Zhang Z, Aihara K, Chen P, Liu R, Chen L. Earthquake alerting based on spatial geodetic data by spatiotemporal information transformation learning. Proc Natl Acad Sci U S A. 2023;120(37):e2302275120.

# [6] Chen P, Suo Y, Aihara K, Li Y, Wu D, Liu R, Chen L. Ultralow-dimensionality reduction for identifying critical transitions by spatial-temporal PCA. Adv Sci. 2025;12(20):2408173.

# [7] Zhong J, Li J, Gu X, Ding D, Ling F, Chen P, Liu R. sPGGM: a sample-perturbed Gaussian graphical model for identifying pre-disease stages and signaling molecules of disease progression. Nat Sci Rev. 2025;nwaf189.

# [8] Zhong J, Tang H, Huang Z, Chai H, Ling F, Chen P, Liu R. Uncovering the pre-deterioration state during disease progression based on sample-specific causality network entropy (SCNE). Research. 2024;7:0368.
